# The impact of biosynthesized ZnO nanoparticles from *Olea europaea* (Common Olive) on *Pseudomonas aeruginosa* growth and biofilm formation

**DOI:** 10.1038/s41598-023-32366-1

**Published:** 2023-03-29

**Authors:** Hafez Al-Momani, Dua’a Al Balawi, Saja Hamed, Borhan Aldeen Albiss, Muna Almasri, Hadeel AlGhawrie, Lujain Ibrahim, Hadeel Al Balawi, Sameer Al Haj Mahmoud, Jeffrey Pearson, Christopher Ward

**Affiliations:** 1 Department of Microbiology, Pathology and Forensic Medicine, Faculty of Medicine, The Hashemite University, P.O box 330127, Zarqa, 13133 Jordan; 2 Faculty of Applied Medical Sciences, The Hashemite University, Zarqa, 13133 Jordan; 3 Department of Pharmaceutics and Pharmaceutical Technology, Faculty of Pharmaceutical Sciences, The Hashemite University, Zarqa, Jordan; 4 Nanotechnology Institute, Jordan University of Science and Technology, P.O. Box 3030, Irbid, 22110 Jordan; 5 Infection Control Unit, King Hussein Cancer Center, Amman, Jordan; 6 Department of Basic Medical Science, Faculty of Medicine, Al- Balqa’ Applied University, AL-Salt, Jordan; 7 Biosciences Institute, Newcastle University Medical School, Newcastle Upon Tyne, NE2 4HH UK; 8 Translational and Clinical Research Institute, Newcastle University Medical School, Newcastle Upon Tyne, NE2 4HH UK

**Keywords:** Cell biology, Microbiology, Molecular biology, Medical research, Nanoscience and technology

## Abstract

There is a limitation in the range of effectual antibiotics due to the *Pseudomonas aeruginosa* (PA) infection due to its innate antimicrobial resistance. Researchers have therefore been concentrating their efforts to discover advanced and cost effective antibacterial agents among the ever-increasing PA bacterial resistance strains. It has been discovered that various nanoparticles can be employed as antimicrobial agents. Here, we evaluated the antibacterial properties of the Zinc Oxide nanoparticles (ZnO NPs), which was biosynthesized, being examined on six hospital strains of PA alongside a reference strain (ATCC 27853). A chemical approach was applied to biosynthesize the ZnO NPs from *Olea europaea* was performed, and confirmed by using X-ray diffraction and Scanning Electron Microscopes. The nanoparticles then applied their antibacterial properties to examine them against six clinically isolated PA strains alongside the reference strain. This process tested for the results of the minimum inhibitory concentration (MIC) and the minimum bactericidal concentration (MBC). The Growth, biofilm formation and eradication were analyzed. The influence of the differentiating degrees ZnO NPs in regard to Quorom sensing gene expression were further examined. The ZnO NPs exhibited a crystalline size and diameter (Dc) of 40–60 nm and both the MIC and MBC tests revealed positive outcomes of concentrations of 3 and 6 mg/ml for each PA strain, respectively. At sub inhibitory concentration, The ZnO NPs were found to significantly inhibit the growth and biofilm formation of all PA strains and decreases in the biomass and metabolic behavior of PA established biofilms; these decreases varied depending on the dosage. At ZnO NPs concentrations of 900 µg/ml, the expression of majority of quorum sensing genes of all strains were significantly reduced, at ZnO NPs concentrations of 300 µg/ml, few genes were significantly impacted. In conclusion, the treatment of PA and could be other antibiotic resistant bacteria can therefore be approached by using ZnO NPs as it has been uncovered that they withhold advanced antibacterial properties.

## Introduction

Microbial colonization, decreased lung capacity, and thick viscous secretions due to malfunctioning ion transfer across the epithelium are all symptoms commonly observed in CF patients^1^. The principal pathogen related to CF is *Pseudomonas aeruginosa (*PA)*.* Although the quality of antimicrobial therapy has been enhanced, PA very difficult to be eliminated once a patient has begun to suffer repeated infections^2^. This is because the tendencies of PA to evade the host’s immune response; and become more resistant to antibiotics, adopting a biofilm mode of growth, with associated production of toxins^3^. Therefore, progressive and eventually fatal reductions in lung function can occur in CF patients who suffer chronic PA infections^3^.

Traditional antibiotics are usually not able to entirely eliminate biofilm infections, due to the unique structure and characteristics of biofilm structures^4^. It is possible for biofilm bacteria to have a 1000-fold higher tolerance of antibiotics than free-floating (planktonic) bacteria^5^. This distinction in antibiotic resistance reveals why patients with biofilm infections are likely to be subject to chronic complications and highlights the need to consider strategies in addition to the use of antibiotics to treat patients in a number of clinical setting, including the management of people with CF^6^.

Nanotechnology has become increasingly recognized as having a potential role in medical science, which includes novel applications relevant to treating infection. The US Food and Drug Administration (FDA) categorizes zinc oxide (ZnO) as “generally recognized as safe” (GRAS)^7^. The antimicrobial behavior of ZnO nanoparticles (NPs) is more evident than that of large ZnO particles; this is because their small size and high surface-to-volume ratio permit increased interaction with bacteria^8^. Studies have proposed that nano-metal oxides, particularly ZnO NPs, can be utilized as disinfectants and antimicrobial agents for nosocomial infections^9,10^. Researchers have investigated the toxicity of ZnO NPs to gram-negative and gram-positive bacterial systems, *Escherichia coli*, *Staphylococcus aureus*, and primary human immune cells^11^. These studies have indicated that it may be possible to employ ZnO NPs as therapeutic antimicrobial agents but there is little data in the area of CF. For our study therefore we have biosynthesized ZnO NPs using *Olea europaea* (common olive) and explored the antibacterial impacts of ZnO NPs on PA isolated from CF patients. The process of biosynthesizing nanoparticles using the plant is both clean and cost effective because plants are readily available and a natural property.

## Methods

### Bacterial isolates and their identifications

The American Type Culture Collection (ATCC) PA 27853 standard strain was used in this study as well as six clinical isolates: PA1, PA2, PA3, PA4, PA5, and PA6. PA 27853 was obtained from the international PA panel; these whole genome sequence is available^12^. The microbiology department at Prince Hamza Hospital, Jordan, and the microbiology laboratory at Jordan University provided the clinical isolates. Samples were obtained from the sputum of CF patients. We identified PA bacteria using Gram-stain; the production of green pigments on nutrient agar; growth on MacConkey agar; and the oxidase test, motility, and growth on selective medium-cetrimide agar; the ability to grow was present at 42 °C. This was verified with the VITEK2 computer automatic bacteria identification system (Bio Merière, Lyon, France).

### Culture conditions

Pseudomonas Cetrimide Agar (OxoidTM) was the selective medium used to isolate PA. A brain–heart infusion (BHI) broth medium (OxoidTM) was employed to enrich the samples. Next, Cetrimide Agar was used to culture the samples, employing the streak plate and pour plate process. Subcultures were initiated from single colonies for each strain and grown on Pseudomonas Cetrimide Agar plates at 37 °C. The samples then underwent aerobic incubation at 37 °C in 5 mL Luria–Bertani (LB) medium. All strains were archived at − 70 °C in an LB medium with 15% glycerol.

### Inoculum of microorganisms

After incubating for 18–24 h, fresh cultures were prepared in a concentration of 0.5 McFarland Scale (1.5 × 10^8^ CFU/mL); different dilutions of these were used in the proposed evaluations.

### Green synthesis of ZnO Nanoparticles

#### Plant extract

In July 2021, leaves were harvested from *Olea europaea* plants growing in the Ibbin area of Ajloun Governorate of Jordan from H Al-momani own farm. To confirm the leaves’ identity, they were compared against the verified sample held in the herbarium of the Faculty of Agriculture at the Jordan Uiversity of Sceince and Technology. Institutional, national, and international guidelines and legislation were observed during the leaf harvesting process.

To prepare the leaves for processing, they were washed in double-distilled water (Sigma-Aldrich), then left at room temperature to dry completely. Once dry, the leaves were placed into an electric grinder and ground to yield a coarse powder. A mixture was made using 5 g of powder and 50 mL of double-distilled water. To obtain the extract, the mixture was heated in a 70° C water bath 15 min. The mixture was then filtered and the recovered extract was stored at − 4° C until required.

#### ZnO nanoparticles synthesis

In a typical procedure, 10 mL of the aqueous yellow leaf extract of *Olea europaea* (common olive) was mixed with 100 mL of 5 mM of aqueous zinc sulfate heptahydrate (ZnSO_4_ · 7H_2_O, ACS reagent, 99%, Sigma-Aldrich, St. Louis, MO, USA) and stirred at room temperature for 10 min to achieve a pale yellow solution. The presence of the polyphenols and other metabolites in the leaf extract play a crucial role in the reduction of the Zn ions and stabilization of the nanoparticles^13^. The plant extract used in the synthesis process also influence size optimization and the yield of NPs.

**Figure 1:**
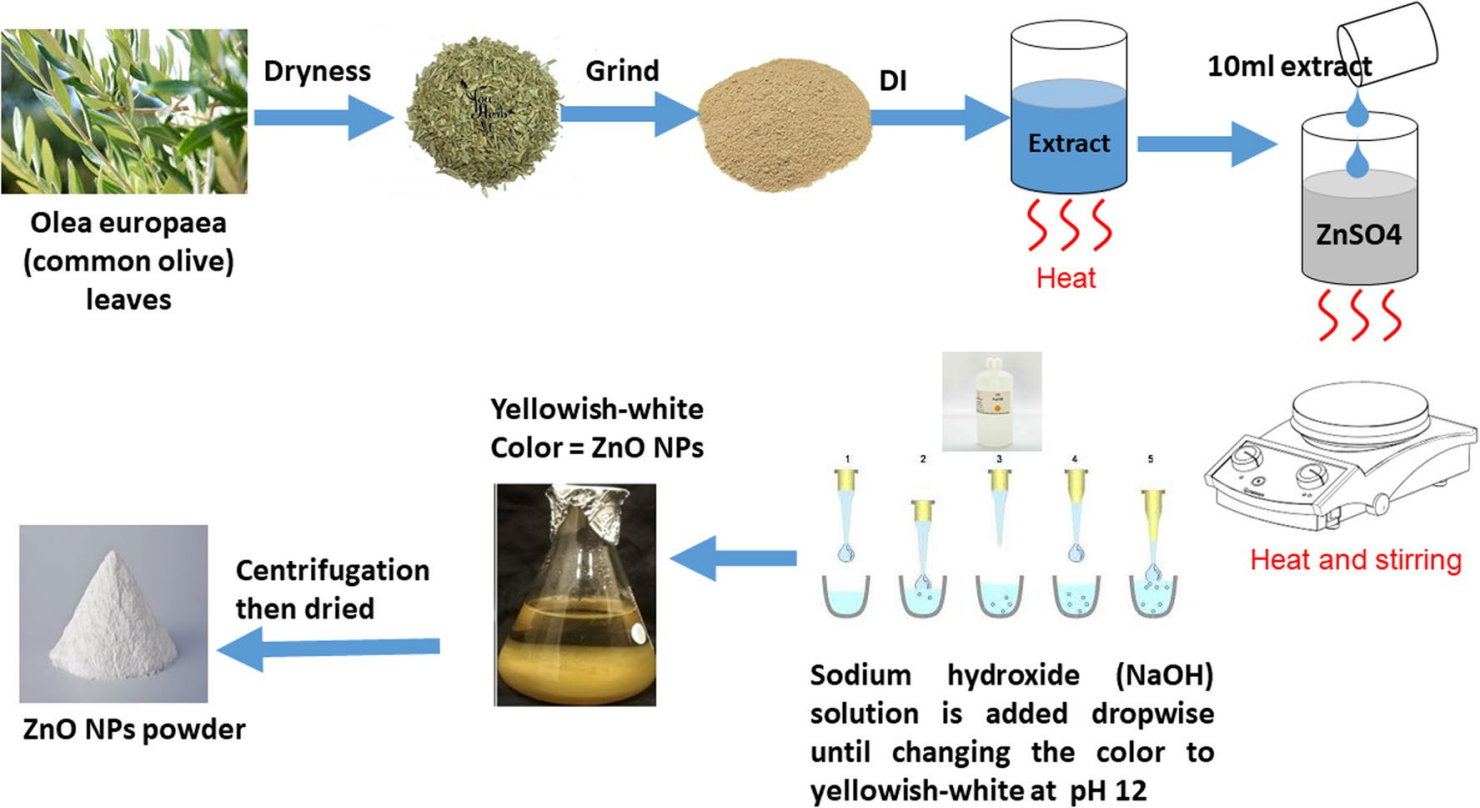
Schematic illustration of the synthesis protocol of ZnO NPs.

After that, 1 M sodium hydroxide (NaOH) solution was added dropwise (4.3 ml of 1 M NaOH over 2–4 h) to the mixture, and stirred at room temperature until the color of the suspension changed to yellowish-white at pH 12. NaOH concentration plays a vital role in determining the morphology, size and optical bandgap of obtained nanostructures^14,15^. The reason for optimizing the base concentration was to produce the desired particle size and shape, which is important as the particle size is directly related to ionic strength and the nucleation of ZnO NPs. Schematic illustration of the synthesis protocol of ZnO NPs is presented in Fig. 1.

The suspended particles were purified by dispersing them with sterile, double- distilled water and centrifuged three times. The resulting white ZnO powder was washed with ethanol to remove the impurities and dried at 80° C in vacuum oven for 5 h to obtain the final product. To obtain the optimum ZnO particle size, the procedure was repeated by using different concentrations of *Olea europea* leaf extract, ZnSO_4_, and 7H_2_O at room temperature.

### Characterization of ZnO NPs

#### Scanning electron microscope

A scanning electron microscope (SEM) was used to evaluate the surface morphology and size of the ZnO NPs. Samples were coated with a fine layer of gold 4 nm and assessed using low vacuum 50 Pa and 3 kV with a working distance of 8–10 mm^16^.

#### X-ray diffraction

The x-ray diffraction (XRD) spectra of ZnO NPs were determined using an x-ray diffractometer (XRD-6000, Shimadzu) equipped with CuKα, a radiation source with a wavelength of 0.154 nm. The sample holder was 2 cm long and 0.5 mm wide.

#### Particle size analyses

Particle size measurements were performed using a Malvern Zetasizer Nano ZS90. The stock suspensions were diluted in distilled water. Particle samples were allowed to equilibrate at 25 °C for 5 min to ensure temperature homogeneity prior to making triplicate tests, each consisting of three individual runs. The viscosity and refractive index values of the solution used were 0.8872 cP and 1.59, respectively.

All of the above mentioned tests were done at the Nanotechnology Institute/ Jordan University of Science and Technology.

### Antibiotic susceptibility testing of the clinical isolates

Assessments of antibiotic susceptibility for the PA strains were conducted with the disc diffusion technique, as described by the Clinical Laboratory and Standards Institute (CLSI), using 10 anti-pseudomonal antibiotics^17^. From the bacterial exponential growth (~ 18–24 h), a bacterial cell suspension in saline was adjusted to 0.5 McFarland units and inoculated in Muller Hinton Agar–MHA (Biolab, Hungary). Antibiotic discs were positioned on the plate and incubated at 35 °C (± 2) for 18–24 h. Bacterial susceptibility to these antibiotics was assessed by calculating the diameter of the inhibition zones that were formed which were then evaluated against values reported by the CLSI^17,18^.

The 10 anti-pseudomonal antibiotics used were as follows: aztreonam 30 µg (ATM), piperacillin 100 µg (PRL), ceftazidime 30 µg (CAZ), cefepime 30 µg (FEP), ciprofloxacin 5 µg (CIP), levofloxacin 5 µg (LEV), amikacin 30 µg (AK), gentamicin 10 µg (CN), meropenem 10 µg (CT), and imipenem 10 µg (IPM). Anti-pseudomonal discs were purchased from TCI, Japan.

### Minimal inhibitory concentration assays

A microtiter broth dilution process that is largely trusted, straightforward, repeatable, inexpensive, and sensitive^19^ was employed to identify the minimum inhibitory concentration (MIC) of the ZnO NPs. The ZnO NPs were used to challenge the ATCC reference PA strain PA 27853 and six clinical isolated PA strains in triplicate in three separate experiments. For each one, 100 μl of bacteria at a density of 5 × 10^5^ CFU/ml in MHB (Biolab, Hungary) were inoculated into the wells of 96-well assay plates (tissue culture-treated polystyrene; Costar 3595, Corning Inc., Corning, NY) with various ZnO NPs concentrations.

The highest concentration of ZnO NPs evaluated in this experiment was 6 mg/ml. Serial double dilution was conducted until the lowest concentration of 23.4 μg/ml was obtained for evaluation. The inoculated microplates were incubated at 37 °C and 150 rpm for 24 hours. Quality control (QC) was performed using PA ATCC 27853. The negative control included solely inoculated broth and was incubated for 24 hours at 37 °C. The lowest ZnO NPs concentration, where there was no visible growth in the wells, was the MIC endpoint.

To verify this, the MIC of ZnO NPs was also calculated by employing a Tetrazolium-based microtiter dilution technique^20^, different tetrazolium-based dyes have been used to discern MIC and assess biofilms^21^. We used triphenyl tetrazolium chloride (TTC) as a metabolic indicator of bacterial viability and biofilm growth. Microplates were incubated at 37 °C for 4 hours after adding 40 μl of 0.2 mg/mL TCC dissolved in deionized water to the wells. The results were scored by visually evaluating the color change in the wells, as assessed in conjunction with the negative and positive controls. Following the MIC determination of the ZnO NPs, aliquots of 50 ul from all wells with no visible bacterial growth were seeded on BHI agar plates and incubated for 24 hours at 37 °C. The minimal bacteriostatic concentration (MBC) endpoint was considered to have been reached when 99.9% of the bacteria had been killed with the lowest concentration of ZnO NPs. This was achieved by evaluating the volume of bacteria present on pre- and post-incubated agar plates.

### Bacterial growth assays

Sterile, untreated, 96-well, flat-bottomed, transparent microtiter plates (BD Falcon) were used to evaluate bacterial growth and biofilm formation with different ZnO NPs concentrations. For the growth assay, standardized suspensions were produced with cultures of all PA strains by using tryptone soy broth (TSB), with 250 µl aliquots for each suspension; these were combined with 20 µl aliquots of the bacteria in suspension with TSB and various ZnO NPs concentrations (100–1000 µg/ml). Every experiment was conducted in triplicate. Both positive and negative control wells were used: positives had only PA and TSB and negatives had only TSB. Samples then underwent aerobic incubation and shaking at 37 °C for 24 h in the dark. The total cell numbers at various ZnO NP concentrations following incubation were calculated by assessing the optical density at 600 nm (OD_600_, Infinite® 200 PRO NanoQuant, TECAN). The value achieved from the readings from wells with *PA* minus the average value of the readings from wells without PA (blank) at the respective ZnO NPs concentration was considered to be the final cell growth value. The total cell growth measured was that for all cells (biofilm-associated and planktonic). In different experiment, TCC was used to assess the impact of ZnO NPs on metabolic activity of PA ug.

### Quantitative determination of biofilm formation via microtiter-plate assessments

Biofilm formation was quantitatively assessed using a spectrophotometric procedure to calculate the total biofilm biomass, counting bacterial cells and EPS. For each condition that was assessed, two wells were prepared on separate, parallel microtiter plates; one was later stained with CV and the other was treated with a metabolic dye TTC. Similar to the method employed for the growth assay, after a 24-h incubation without shaking, the well aspirates were thrice washed in sterile Phosphate Buffered Saline (PBS) (250 µl). The plates were then forcefully shaking to eliminate any bacteria that had not adhered to the bottom of the wells.

For the first plate, the microbes that remained were fixed using 99% methanol at 200 µl for each well. The first plates were left for 15 min before being emptied and allowed to dry. The plate was then stained for 5 min using 2% Hucker crystal violet suitable for Gram-stain use at 200 µl for each well. Any surplus staining material was removed and the wells were thrice washed in 200 μL of sterile water, being careful not to dislodge the biofilm. The plates were then again left to dry and resolubilization of the cell-bound dye was achieved using 33% (v/v) glacial acetic acid at 160 µl for each well.

Optical density (OD) was calculated for all wells using Infinite® 200 PRO NanoQuant, TECAN, an automated reader. Measurements were read at three points: (1) before the samples were incubated (OD 600 nm), (2) after the samples had been incubated and were at the growth assessment stage (OD 600 nm), and (3) after the biofilm assay had been produced (OD 570 nm). The ratio chosen for normalization of the calculation of biofilm that appeared compared to the amount of bacteria growth was 570:600. A negative OD value was displayed as 0. All tests were conducted thrice in triplicate. A cut-off value (ODc) was confirmed: three standard deviations (SD) above the mean OD of the negative control: Odc = average OD of negative control + (3 × SD of negative control). The isolates were placed into one of four groups according to the OD: non-biofilm producer (OD < ODc), weak-biofilm producer (ODc < OD < 2 × ODc), moderate-biofilm producer (2 × ODc < OD < 4 × ODc), and strong-biofilm producer (4 × ODc < OD).

The metabolic activity for the second plate was also assessed. TTC was employed as an indicator of viable bacteria^22^. Cells characterized by significant metabolic activity transform TTC into a colored formazan derivative; this colored derivative can then be assessed to measure PA*-*viable cells. Similar to the procedure for the growth assay, after a 24-h incubation period at 37 °C, the plates were forcefully shaken to eliminate any bacteria that had not adhered to the plate. Media was removed from all wells from all wells following the incubation periods. The biofilm that had appeared was washed once in 200 μL of phosphate-buffered saline (PBS). Then, the wells containing biofilm were combined with 100 μL of PBS solution and the biofilm cells were suspended through forceful pipetting. The suspended biofilm was moved to a new 96-well flat-bottomed microplate. Next, 50 μl of 0.1% TTC (Sigma, USA) was added to a final concentration of 0.02%. After a 2–4-h incubation period at 37 °C, the OD540 was measured. Since the decrease of TTC due to viable bacteria forms red formazan, the reduction of bacterial growth can be calculated quantitatively through colorimetric absorbance at 540 nm (Knezevic and Petrovic, 2008).

### Microtiter biofilm eradication assay

To assess the impacts of ZnO on pre-formed biofilms, a process using a combination of CV staining and a TTC dye was employed. To create biofilms, standardized bacteria suspensions from each PA strain were prepared from overnight cultures using TSB at a concentration of 0.5 McFarland Scale on 2 different 96 well plates and 20 µl aliquots of bacteria suspension were added to 250 µl aliquots of TSB that did not include ZnO NPs. To arrive at a final volume of 270 ul in the microtiter well, the plates were incubated overnight at 37 °C to promote biofilm attachment and growth.

For each concentration that was evaluated, two wells were prepared on separate, parallel microtiter plates: one was later stained with CV and the other was treated with a metabolic dye TTC. The following day, the media was removed and the plate was dried in an upside-down position on a sterile paper towel for 15 min at room temperature. The planktonic and unbound cells were aspirated from each well and the biofilm that remained was thrice rinsed in 150 ul of fresh sterile medium using a multi-channel pipette. Excess rinse material was removed from all wells via aspiration and 200 μl of fresh MHB with different concentrations of ZnO NPs were added at concentration range from 100 to 1000 µg/ml. The plates then underwent overnight incubation at 37 °C. The next day, the planktonic growth was calculated and the planktonic cells and the media were discarded; the biomass that was left was thrice rinsed in distilled water. The first plate was stained with CV and quantified using the method described above for the biofilm assay. The second plate was stained with TCC.

The amount of biofilm inhibition was measured in relation to the media sterility control (defined as 0%) and the amount of biofilm growth in the absence of ZnO NPs (defined as 100%). The average of the results from three separate biological replicates was calculated. The mean OD600 values for the biofilms following treatment were expressed as a percentage in relation to the control.

### Gene expression tests associated with the construction of biofilm

To calculate the impact of different concentration of ZnO NPs on the relative gene expression of QS-regulatory genes, qRT-PCR was used, the total bacterial RNA was extracted at the middle of the log phase, corresponding to OD600 of 0.5–0.6, from PA subjected to a fairly low concentration (300 µg/ml) and high level (900 µg/ml) of ZnO NPs dispersed in the the MH medium. The controls without ZnO NPs were treated in the same manner. After exposure for 24–48 h (exponential stage of biofilm formation), the biofilm was carefully collected and washed in 10 mM NaCl to eliminate any unbound cells. An RNeasy Mini Kit (Qiagen, , Germany) was used to extract the biofilm RNA and the RNA concentration was calculated in ng/μL with a Nanodrop ND-1000 tool (Nanodrop Products Inc., Wilmington, NE) that also verified the RNA purity at absorbance (260/280 nm).

We synthesized cDNA at 42 °C via reverse transcription polymerase chain reaction (PCR) with random primers, RNaseOUT, dNTPs, and Superscript II reverse transcriptase (EasyScript, transgenbiotech, China). Quantitative polymerase chain reaction (qPCR) was conducted with a BIO-RAD Thermal cycler; for amplification, 2 μL of template DNA was combined with 0.5 μL of each forward and reverse primer, 10 μL of Luna Universal qPCR Master Mix, and 7 μL of nuclease-free water. The resultant reaction volume was 20 μL. The Primer sequences used are presented in Table S1.

A gradient PCR reaction was conducted for all PCR primers. The cycling conditions included pre-denaturation at 95 °C for 3 min and then 34 cycles of denaturation at 94 °C for 30 s, annealing with a temperature range between 50 and 63 °C for 30 s, elongating at 72 °C for 1 min, and extending at 72 °C for 5 min. The relative expression values of QS-regulatory genes were normalized to the housekeeping gene *rpoD* and agarose gel electrophoresis was employed to verify the specific level of PCR amplification. The relative gene expression in cultures treated with ZnO NPs was analyzed alongside an analysis of their expression levels in cultures with no ZnO NPs according to the 2-∆∆Ct procedure.

### Ethical approval

Hashemite University and Prince Hamza Hospital’s Ethics Service Committee granted ethical approval for this case study (reference number 7/10/2019-2020). All experiments were performed in accordance with relevant guidelines and regulations. PA clinical isolate form sputum sample were taken after an informed consent was obtained from all CF patients.

### Statistical analysis

The results from the tests were summarised as mean ± standard error of mean of at least three replicates. Where relevant, distinctions between samples and controls were evaluated via one-way analysis of variance (ANOVA). The OD values from the microtiter plate experiments both with and without treatment with ZnO NPs at various dilutions were compared using Tukey’s test. *P* values of < 0.05 were taken to be significant. Data were analyzed using Graphpad Instat 6.0 software.

## Results

### X-Ray diffraction, SEM and particle size analyses

**Figure 2:**
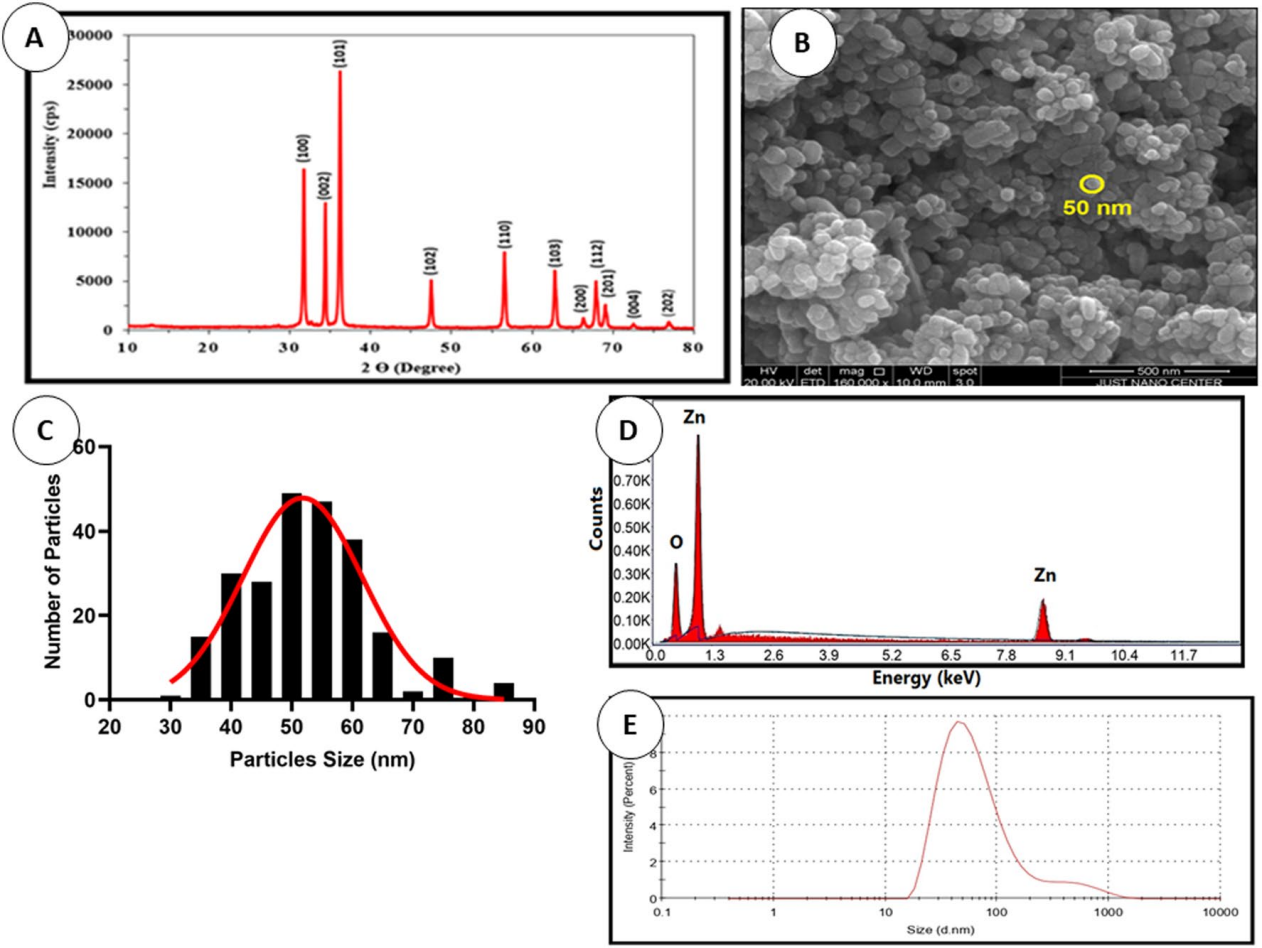
(**A**) XRD- pattern indicating presence of ZnO NPs peaks, (**B**) SEM image SEM images of ZnO particles showing their morphology captured at 16,000 × magnification, yellow circles indicate the NPs circumference, (**C**) Particle size distribution from SEM results, (**D**) EDX Spectra of Zinc Oxide Nanoparticles, and (**E**) Particle size distribution indicating average size of ZnO NPs.

XRD studied the crystalline phases formation of synthesized ZnO nanoparticles using CuKα radiation of wavelength 1.5406 Å in the range of (2θ) from 10° to 80° (at room temperature of 298 K). Figure 2A showed that the pure sample of ZnO-NPs demonstrate the hexagonal wurtzite ZnO structure corresponding to the crystal planes (of given Miller indices h, k and l): (100), (002), (101), (102), (110), (103), (200), (112), (201), (004), (202). This indicates that the crystals are quite pure with no traces of any other impurities in the crystal structure. To have a clear idea about the crystal properties of the ZnO NPs, the crystal size was calculated using the well-known Debye-Scherrers formula based on the major diffraction peaks, mainly (101) with the highest peak intensity, and it was found to be of around 42 nm. In this regard, this method of particle- size calculation is not quite suitable for particles at the nanoscale because it depends on the, broadening of the XRD diffraction peaks, crystallite size, and the detection limit of the diffractometer. The crystallite size here is assumed to be the size of a coherently diffracting domain and it is not necessarily the same as the average particle size. Moreover, the maximum or upper-limits strain (ε_hkl_) in the ZnO crystals was calculated based on the ratio of the difference between the ideal (d_0_) and observed (d) inter-planner spacing to the ideal value for the major diffraction peaks (ε_hkl_ = (d_0 _− d)_hkl_/(d_0_)_hkl_). For example, a good estimation of the strain for the (101) peak was found to be ε_101_ = 0.005. As for the average dislocation density (δ), which is inversely proportional to the particle size(D) (i.e. δ = 1/D^2^), was found to be of about 5.66 × 10^−4^ nm^−2^. These results indicate that the ZnO crystals are of quite good crystalline properties with almost perfect crystal structure.

The morphological features of ZnO NPs were conducted using SEM. Samples were sputtered with a fine layer of gold 4 nm and assessed using low vacuum 50 Pa and 30 kV with a working distance of 8.5 mm. SEM images revealed the formation of homogeneous shape and size ZnO nanoparticles with an average size of 50 nm as shown in Fig. 2B. The size histograms of the ZnO-NPs are shown in Fig. 2C. The histograms indicate that the main particle sizes of the ZnO-NPs made in this research is about 52.8 ± 10.6 nm. Moreover, the EDX result for the selected scanned area of the SEM image is shown in Fig. 2D. To have a clear idea about the statistical particle-size distribution and the percentage of occurrence of the particles, several particle-size measurements were conducted and shown in Fig. 2E. These results are in good agreement with the results obtained from the SEM and XRD techniques. Even though, some low traces of particles with larger sizes were observed in the size distribution curve.

### Antibiotic susceptibility and resistance patterns of clinical isolates

The disc diffusion process was employed to test antibiotic susceptibility. Two clinical isolates (PA3 and PA5) were resistant to Cefepime and three clinical isolates (PA2, PA4, and PA5) had an intermediate resistance to one, two, and four antibiotic used in this experiment, respectively. They were no multi-drug resistant (MDR) PA. The full results of the experiment to determine susceptibility to antibiotics are shown in Table S2. PA5 had the strongest resistance to the antibiotics used in this experiment.

### MIC and MBC

After a 24-h incubation period in aerobic conditions at 37 °C, turbidity was observed in the test tubes with 23.4 μg/ml–1.5 mg/ml ZnO NP concentrations in all PA strains, indicating bacterial growth. In test tubes with ZnO NP concentrations of 3 and 6 mg/ml, no turbidity was observed among all tested strains, indicating the inhibition of bacterial growth. The suspension from the tubes of 3 and 6 mg/ml was inoculated in a BHI agar plate and incubated for 24 h. There was no bacterial growth in the plate with 6 mg/ml, showing that it was bactericidal. Therefore, these results demonstrate that the MIC and MBC of ZnO NPs for all PA strains were successful at concentrations of 3 and 6 mg/ml, respectively.

### Inhibitory effect of ZnO NPs on planktonic growth and biofilm formation of *P. aeruginosa*

**Figure 3:**
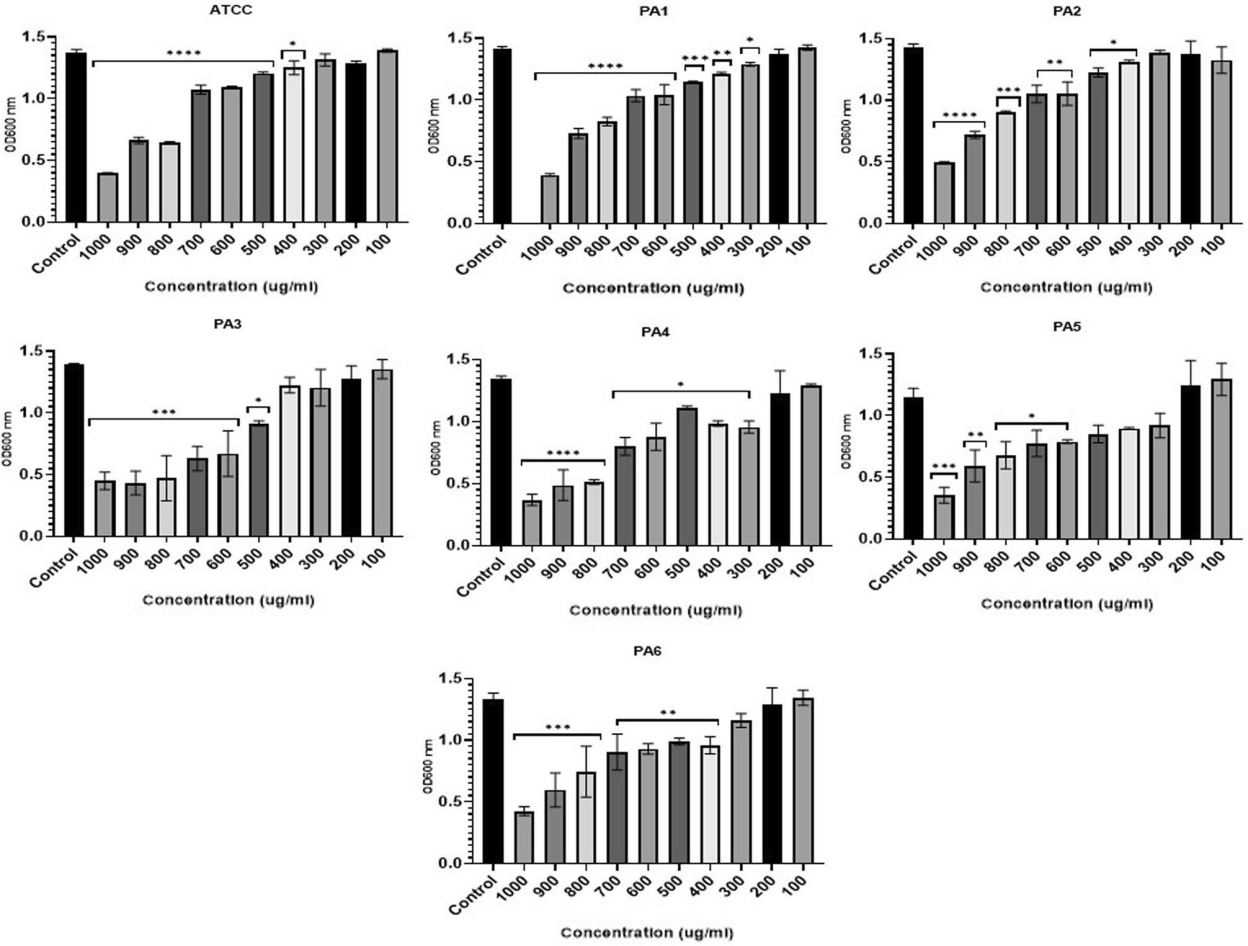
The effect of ZnO NPs on growth of PA represented by OD at 600 nm (y-axis) for the ATCC strain and six clinically isolated strains (PA1-PA6) at different concentrations of ZnO NPs range from 100 to 1000 µg/ml (x-axis) after 24-h incubation period. **** < 0.0001, ***0.0001, ** < 0.001, * < 0.01.

The microtiter plate assay results showed that all strains were strong biofilm producers (Table S3). Incubating PA strains with ZnO NP concentrations of 100–1000 µg/ml negatively affected growth, however, the results varied between strains with statistically significant inhibitory effect at high concentration detected by performin on way ANOVA (F (2.013, 12.08) = 61.93, *P* < 0.0001) (Fig. 3). ZnO NPs Concentrations 400 to 1000 µg/ml significantly inhibited growth (*p* < 0.05) in three PA strains (ATCC, PA2, and PA6). Strains PA3 and PA5 were significantly impacted by concentrations of 500 and 600 µg/ml and above, respectively. Concentrations of 300–1000 µg/ml inhibited the growth of PA1 and PA4 at statistically significant level. ZnO NPs in concentrations of 100–200 µg/ml negatively affected growth for all strains, however, this was not statistically significant (Fig. 3).

**Figure 4:**
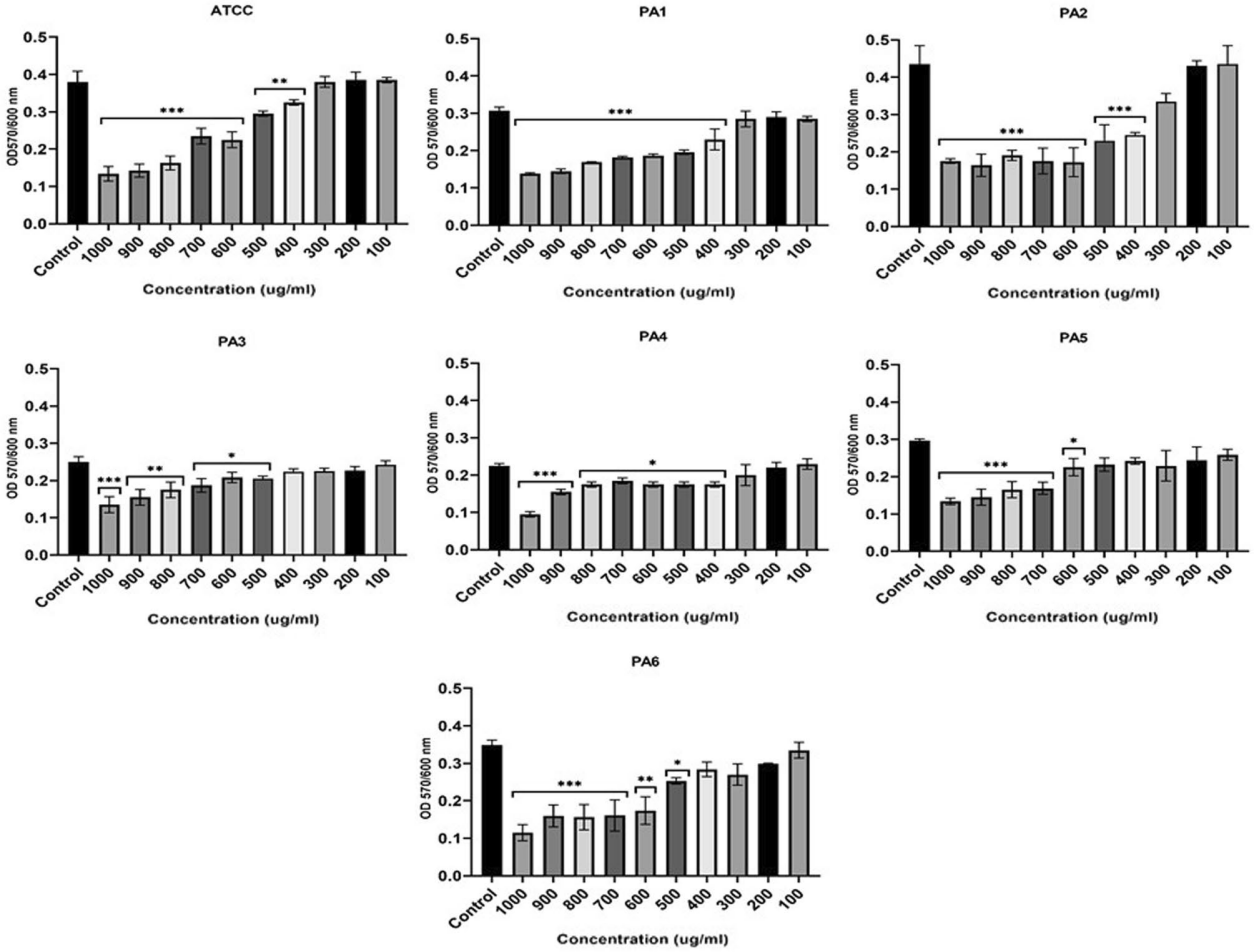
The effect of ZnO NPs on biofilm formation represented by OD at 570/600 nm (y-axis) for the ATCC strain and six clinically isolated strains (PA1-PA6) at different concentrations of ZnO NPs range from 100 to 1000 µg/ml (x-axis) after 24-h incubation period. **** < 0.0001, ***0.0001, ** < 0.001, * < 0.01.

The biofilm formation in four strains (ATCC, PA1, PA2, and PA4) was significantly impacted (ANOVA test: F (1.731, 10.39) = 16.12, *P* = 0.0009) by ZnO NPs concentrations of 400 to 1000 µg/ml. biofilm formation of strain PA3 and PA6 were statistically significantly inhibited impacted with ZnO NPs concentrations of 500 to 1000 µg/ml. The most antibiotic resistant strain of PA evaluated in our experiments, PA5, was impacted with ZnO NPs concentrations of 600–1000 µg/ml (Fig. 4).

**Figure 5:**
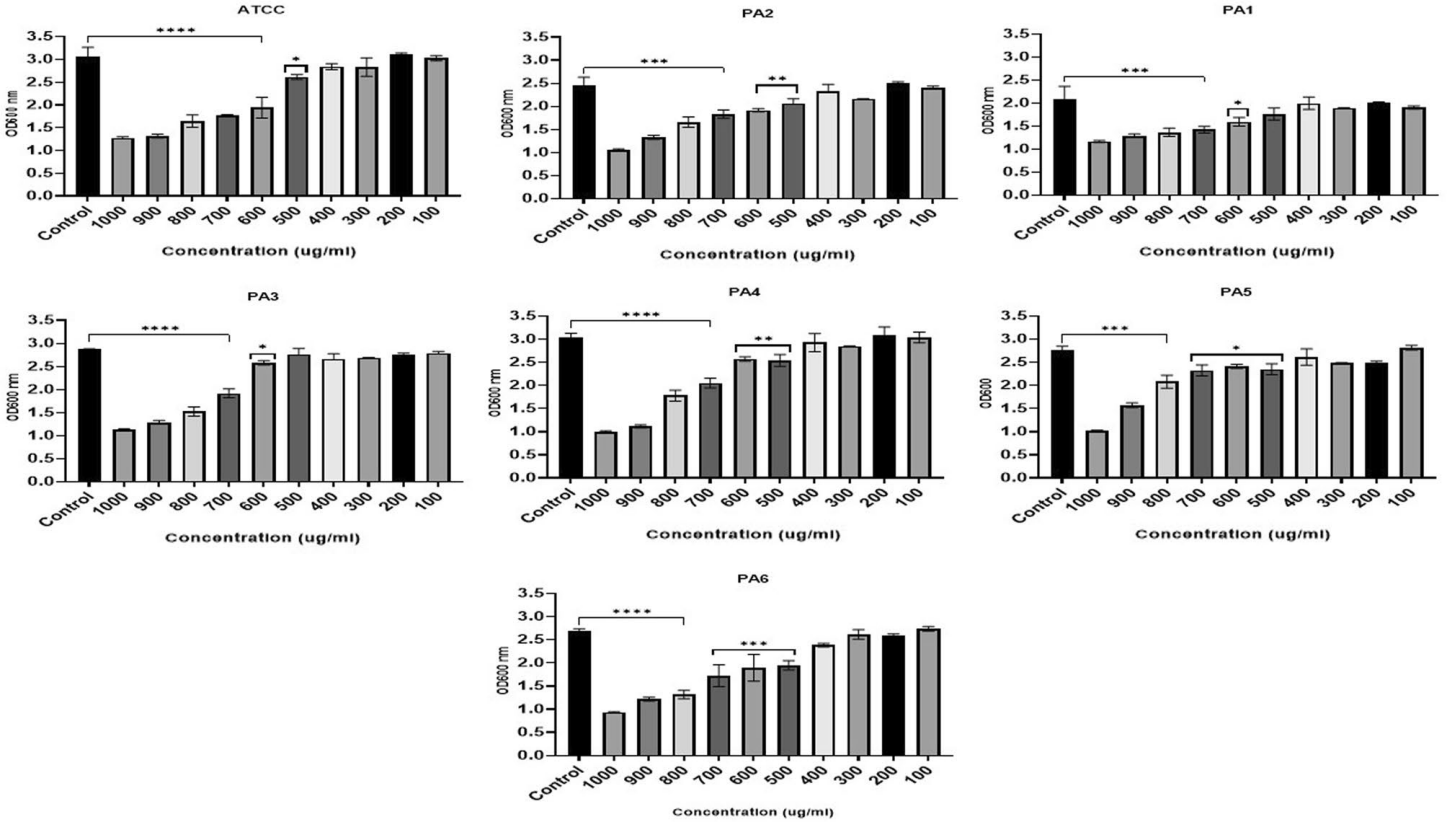
The effect of ZnO NPs on metabolic activity of PA blanktonic cells represented by OD at 600 nm (y-axis) after adding TCC strain for 4 h for the ATCC strain and six clinically isolated strains (PA1-PA6) at different concentrations of ZnO NPs range from 100 to 1000 µg/ml (x-axis) after 24-h incubation period. **** < 0.0001, ***0.0001, ** < 0.001, * < 0.01.

There was a statistically significant reduction in the metabolic activity of PA with biofilm compared to the control (F (2.657, 15.94) = 43.30, *P* < 0.0001). The metabolic behavior of the biofilm indicated that a ZnO NPs concentration of 500 to 1000 µg/ml significsntly reduced the metabolic behavior of biofilm cells in ATCC, PA2, PA4, PA5, and PA6. ZnO NPs concentrations of 600–1000 µg/ml greatly impacted PA1 and PA3, although, all ZnO NPs concentrations negatively affected the metabolic behavior of biofilm cells (Fig. 5).

### Impact of ZnO NPs on preformed biofilm

**Figure 6:**
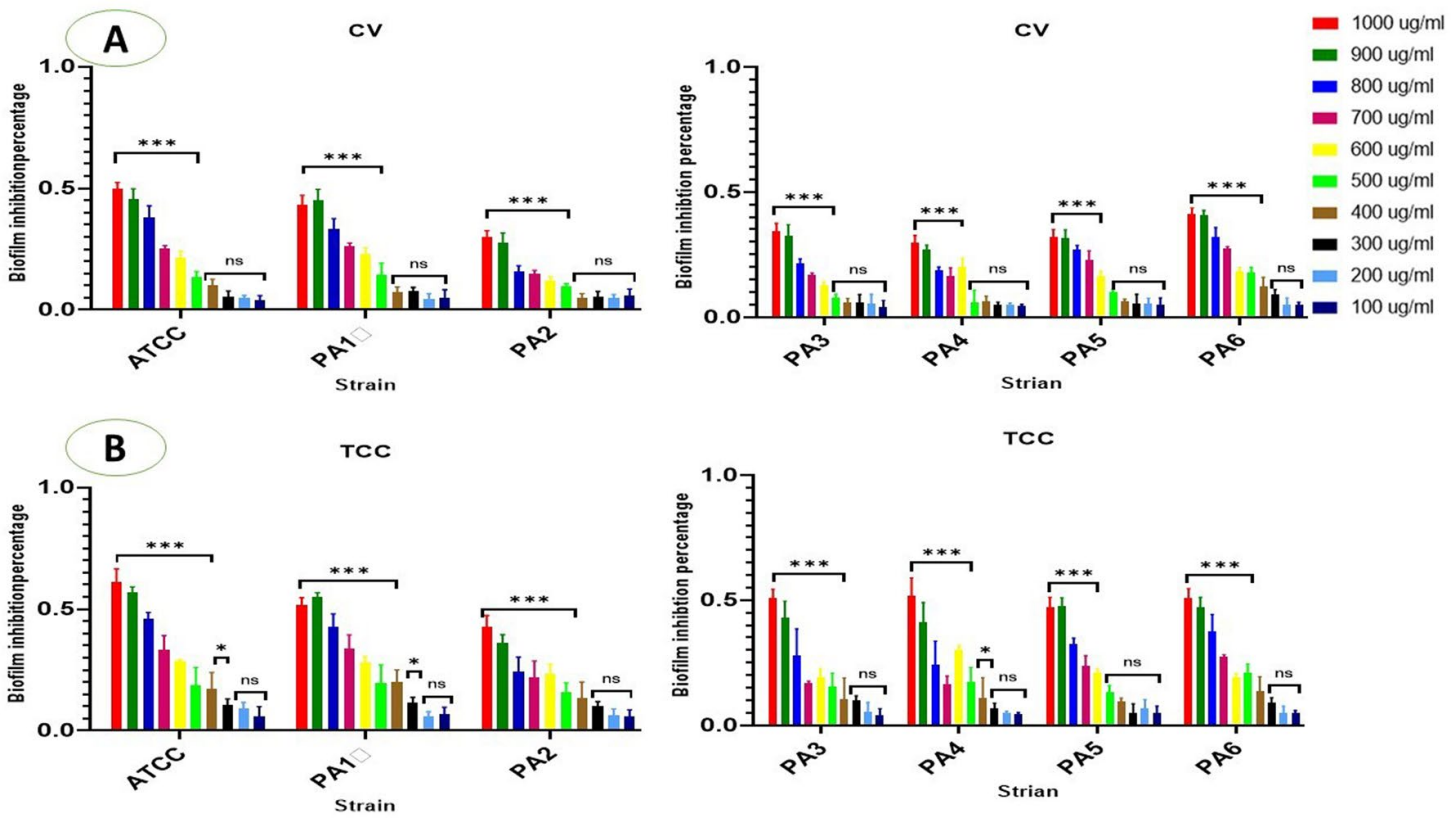
(**A**) The percentage of mature biofilm inhibition after 24 h incubation with ZnO NPs at concentration 100–1000 µg/ml, y-axis represent the percentage of mature biofilm eradication and the x axis represent the PA strain after CV staining. (**B**) The percentage of mature biofilm metabolic activity after 24 h incubation with ZnO NPs at concentration 100–1000 µg/ml, y-axis represent the percentage of percentage of biofilm metabolic activity and the x axis represent the PA strain after CV staining and TCC staining **** < 0.0001, ***0.0001, ** < 0.001, * < 0.01, ns: not statistically significant.

Regarding the eradication assays that were used to evaluate the treatment of 1-day-old pre-formed biofilms. There was an overall reduction in biofilm biomass in all strain exposed to ZnO NPs compared to control (F (20, 66) = 9.148, *P* < 0.0001). However, this impact was varying according to concentration of ZnO NPs utilized. Concentrations of 500–1000 µg/ml significantly decreased CV staining PA biomass in three strains (ATCC, PA1, and PA2). Concentrations of 600–1000 µg/ml also decreased CV staining in three strains (PA3, PA4, and PA5). Lower concentrations of 300–1000 µg/ml decreased CV staining in one strain (PA6) at statistically significant level (Fig. 6A).

In addition, there were declines in metabolic activity of 1-day old PA biofilm at concentrations lower than those necessary to inhibit biomass. A ZnO NPs concentration of 300 µg/ml greatly decreased metabolic behavior in strains ATCC, PA1, and PA4; ZnO NPs concentrations of 400 µg/ml and above greatly decreased the metabolic behavior of strains PA2, PA3, and PA6. ZnO NPs concentrations of 600 µg/ml and above greatly decreased the metabolic behavior of strain PA5 (Fig. 6B). The planktonic growth of 1-day old biofilm also significantly inhibited after 24-h incubation with ZnO NPs at concentration equal or over 500 µg/ml (Supplementary Fig. 1).

### QS-regulated gene expression

The relative expression of QS-regulated genes *lasI*, *lasR*, *rhlI*, *rhlR*, *pqsR*, and *pqsA* was assessed by referring to the C_*t*_ values. Average relative amounts of tested genes were then normalized to the average relative amount of the *ropD* reference gene in the same sample.

**Figure 7:**
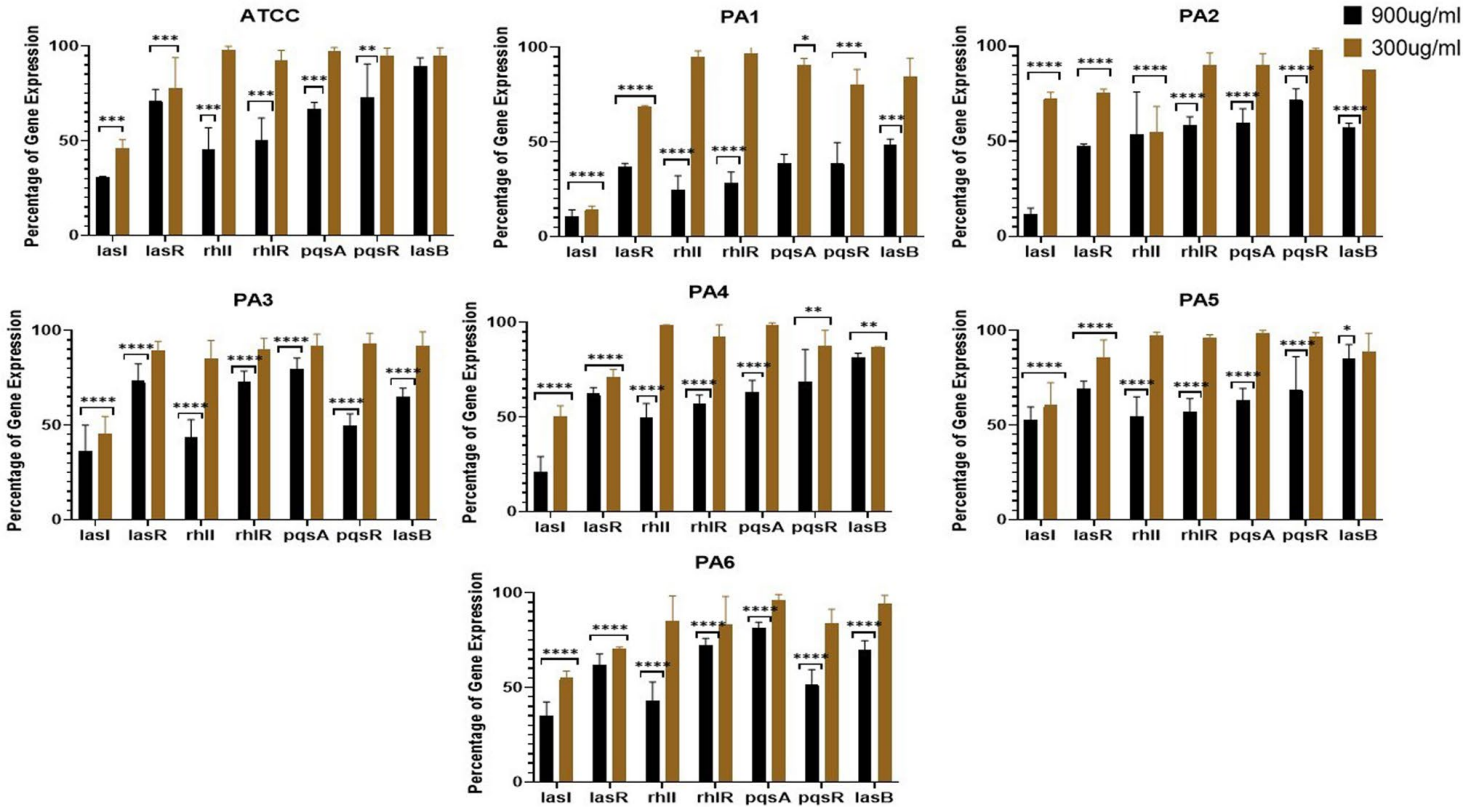
The effect of ZnO NPs at concentrations of 900 and 300 µg/ml on relative expression of QS-regulated genes in PA strain exposed to biosynthesized ZnO NPs compared to the gene expression level of control samples that is not exposed to ZnO NPs.

The relative expression of QS-regulated genes presented in PA strain exposed to ZnO NPs in comparison with their expression in the control were evaluated using the ∆∆Ct method^23^. Changes in expression levels are shown in Fig. 7. The relative expression of the *lasI* gene was greatly reduced from 100% in untreated isolates to 69, 89.3, 88.3, 63.5, 78.8, 47.4, and 64.8% in ATCC, PA1, PA2, PA3, PA4, PA5, and PA6, respectively, in isolates combined with 900 µg/ml concentrations of ZnO NPs. It also decreased from 100% to 53.8, 85.5, 27.2, 54.4, 49.9, 39.5, and 45.1% in in ATCC, PA1, PA2, PA3, PA4, PA5, and PA6, respectively, in isolates combined with 300 µg/ml concentrations of ZnO NPs. The *rhII* gene was the second most effected gene with 900 µg/ml concentrations of ZnO NPs, reducing expression from 100% to 54.3, 75.2, 46.4, 56.5, 50.3, 45.3, and 57% in ATCC, PA1, PA2, PA3, PA4, PA5, and PA6. There were varying degrees of reduction in different genes and strains, however, all genes at all strains showed some decline in gene expression (Fig. 7).

At ZnO NPs concentrations of 900 µg/ml, the expression of all genes of all strains was greatly decreased aside from the *LasB* gene of the ATCC strain; although this gene was also affected, the impact was not statistically significant at a decrease of less than 10%. At ZnO NPs concentrations of 300 µg/ml, few genes were greatly impacted. A great reduction in gene expression was observed for *LasI* in all strains, *LasR* in all strains except PA3, *rhII* in PA2, and *pqsR* and *LasB* in PA4 (Fig. 7).

Supplementary Fig. 2 show the difference in∆∆Ct among different gens. ∆∆Ct refers to the difference between the ∆Ct values of the samples treated with ZnO NPs (experimental) and those not exposed to ZnO NPs (control). A higher ∆∆Ct value indicated a lower gene expression. A ZnO NPs concentration of 900 µg/ml significantly affected the ∆Ct value in all strains and nearly all genes, aside from *lasB* in ATCC and PA5, *pqsR* in PA2, and *pqsA* in PA6. At ZnO NPs concentrations of 300 µg/ml, few genes were greatly affected; these were LasI in all strains, LasR in all strains except PA3, *pqsR* in PA1; and *rhII* in PA2 and PA4.

## Discussion

PA is a principal etiologic agent of nosocomial infections and is particularly relevant to the pathophysiology of chronic lung diseases, including the morbidity and mortality associated with CF lung disease. Thus, effective regulations concerning infection control are urgently needed to counter its spread^24^. PA displays both natural resistance and MDR to antimicrobial elements^25^. To counter the greater tolerance of bacteria to antibiotics, biosynthesized ZnO NPs that can be used to treat biofilm infections may represent a novel strategy relevant to the global challenge of Antimicrobial Resistance and antibiotic stewardship.

This study presents novel data which showed that ZnO NPs that biosynthesized from *Olea europaea* (common olive) were able to broadly antagonize growth of PA, and that importantly this included measurable effects on biofilm mode growth in both lab reference and clinical isolates of PA. ZnO NPs toxicity has been researched in relation to various bacteria, such as *Escherichia coli*^26^, *Salmonella typhimurium*^27^, *Listeria monocytogenes*^28^, *Staphylococcus aureus*^29^, and PA^30^. However, the majority of studies have concentrated on planktonic bacteria. The relationships between NPs and biofilms are not fully known and the impact of NPs on bacterial cell activity and molecular mechanisms is not understood. We therefore assessed the ability of ZnO NPs to reduce or obstruct the growth of a preformed biofilm established on a microtiter plate.

The antimicrobial and antibiofilm behavior of different concentrations of ZnO NPs were assessed concerning both inhibtion (ZnO NPs added before biofilm production) and elimination (ZnO NPs added after biofilm production) in relation to a PA ATCC strain and six clinically isolated strains. For both assays, we evaluated both remaining biomass through CV staining and the metabolic activity of the biofilm sample using TTC dye. TTC is colorless when oxidized and red when decreased by microorganisms due to the production of formazan. Live microorganisms decrease TTC via enzymatic action, producing red formazan that remains inside granules in the cells. This improved our ability to interpret the results^31^.

In this study, ZnO NPs inhibit the growth of all PA isolates at 2.5 mg/ml. When we investigated the impact of lower concentrations of ZnO NPs (100–1000 µg/ml) on microbial growth, we found a statistically significant difference between cultures exposed to ZnO NPs and control cultures. A concentration-dependent inhibtion of growth was identified. This is consistent with the results of previous studies that reported the inhibtion of PA growth in complex liquid media or on agar media by commercial ZnO NPs^32^ and through biosynthesizing ZnO NPs using the seed extract of *Butea monsoperma*^33^.

The Comparisons between our results and previously literature were slightly hindered as the ZnO NPs had different characteristics^34,35^. This may offer a partial explanation concerning why data reported in the literature regarding the MIC of ZnO NPs for various PA strains differ. For example, one study reported a MIC value of ZnO NPs (biosynthesized from *Butea monosperma* ) against standard PAO1 as 1600 µg/mL, whereas the MIC value ranged between 1600 and 3200 µg/mL for clinical isolates derived from various sources^33^. Moreover, The antibacterial activity of ZnO NPs is strongly influenced by concentration and size of the the NPs^36^. Some studies report a direct correlation between the concentration of ZnO NPs and their antibacterial activity^36,37^. The antibacteria activity of ZnO NPs is increase with a larger particles’ surface area^38^. The antibacterial efficacy of ZnO NPs is assisted by the ability of small sized particles being able to penetrate bacterial membranes^37^. Multiple studies have explored the relationship between antibacterial activity and paticle size. These studies report that to maximize bactericidal effects, it was essential to regulate the size of ZnO NPs, and to produce small NPs that have the greatest surface area^37–39^. Partical size has also been identified as determining the dissolution of ZnO NPs into Zn^2+^; some researchers conclude that the toxicity of ZnO NPs is due to the dissolution of Zn^2+^^40,41^. The studies conducted by Padmavathy and Vijayaraghavan^42^, and Guo et al.^43^, analyzed the effect that the size and concentration of ZnO NP has upon the generation of H_2_O_2_; the surface area of ZnO was found to be the key factor. The antibacterial activity of smaller particles is attributed to the increased concentration of oxygen species on the particle surface due to the greater surface area^43^.

Planktonic bacteria were impacted by similar or lower ZnO NP concentrations compared with bacteria in biofilm growth, demonstrating the protective impact of the biofilm formation of PA. A study by Choi et al.^44^ indicated that for the inhibition of *E. coli* biofilms, a higher concentration of sliver NPs was necessary compared to that required for planktonic bacteria. Furthermore, it has been found that biofilms are significantly more resistant to antimicrobial materials than planktonic cells^45,46^. The same is true for toxic metals^47^. Bacteria in biofilms have bioactivities, metabolic pathways, and stress responses distinctive from those of planktonic cells^48^. Research has indicated that a variety of microbes are vulnerable to toxic compounds, including antibiotics, in planktonic media or early biofilm phases but demonstrate a higher tolerance of toxicants in aged biofilms ^49^.

There is little research on the impact of ZnO NPs on established biofilms. We found that ZnO NPs could inhibit the growth of both established biofilms and planktonic bacteria. The concentrations required for this result were higher than those required to inhibit biofilm formation, although the treatments were conducted in identical environments. The US Food and Drug Administration (FDA) categorizes zinc oxide (ZnO) as “generally recognized as safe”^7^.

Biofilms can bind ions to EPS and cell walls; therefore, they can reduce the toxic effects matrix^50^. A study by Joshi et al.^51^ indicated that EPS protected *E. coli* from silver and ZnO NPs and suggested that this protective impact was caused by the accumulation of NPs in the biofilm. Other studies have suggested that there may be an additional protective impact in anaerobic areas of biofilms (anaerobic conditions prevent ZnO NPs from releasing zinc ions). These types of conditions are common in biofilms and the presence of anaerobic microdomains in biofilms has been demonstrated (Lawrence et al. 2007).

QS molecules are important in PA biofilm production and conservation, which is necessary for bacterial adhesion^52^ and we have previously demonstrated QS present in lung allograft recipients who are known to be vulnerable to infection^53^. The presence of QS was shown to impact biofilm production in PA when it was discovered that a *lasI* mutant forms a fine biofilm that is more vulnerable to disturbance by detergents^54^.

To investigate the possible quorum quenching impact of ZnO NPs, the relative expression of QS-regulatory genes that control biofilm production in PA were therefore evaluated via qRT-PCR. We found that the expression of the QS-regulated genes *lasI*, *lasR*, *rhlI*, *rhlR*, *pqsR*, *and pqsA* decreased following exposure to ZnO NPs at concentrations of 900 µg/ml. Abdelraheem and Mohamed^55^ found that ZnO NPs greatly down-regulated biofilm and virulence gene expression in PA clinical isolates for all genes studied aside from the *toxA* gene, which was up-regulated. The fold change reduction in the quorum sensing genes (*LasR*, *rhlI*, and *pqsR*) following exposure to ZnO NPs were 10.4-, 6.3-, and 8.7-fold, respectively^55^. The impact of ZnO NPs is possibly similar to that of silver NPs as both reduce the expression of *lasR* and *rhlR*, causing a disturbance in QS circuits and an ensuing decline in biofilm formation^56^. The molecular basis and complete mechanism of the impact of NPs as quorum sensing inhibitors must be explored further in future studies.

The antibacterial mechanism of ZnO NPs is not yet understood, however, some studies have found the potential for membrane harm due to the direct or electrostatic interactions between ZnO NPs and cell surfaces, cellular internalization of ZnO NPs, and the formation of active oxygen species caused by metal oxides^57,58^. Other research has indicated that the predominant cause of the antibacterial mechanism could be due to the disturbance of cell membrane activity^59,60^. However, Modi et al.^61^ stated that gram-negative antibiotic-resistant bacteria, for example, *Klebsiella pneumoniae* and *Escherichia coli*, have low sensitivity to colloidal ZnO NPs compared to gram-positive antibiotic-resistant bacteria. As previously discussed, the production of intercellular reactive oxygen species, such as hydrogen peroxide, an oxidizing element that harms bacterial cells, may also cause this mechanism^62^. ZnO NPs may also be activated by UV and visible light to generate highly reactive oxygen species, for example, the superoxide anion (O_2_‒), hydrogen peroxide (H_2_O_2_) and the hydroxyl radical (HO^-^). Negatively charged hydroxyl radicals and super-oxides cannot infiltrate the cell membrane and it is thus probable that they will stay on the cell surface, whereas H_2_O_2_ can infiltrate bacterial cells^63^. Other processes that have been suggested to explain the antibacterial impacts of ZnO NPs include functional harm caused by the rough surface of NPs^64^ and the cellular internalization of NPs, which can deform cell walls and cause NPs to bioaccumulate^65^. Furthermore, we have found that ZnO NPs decrease metabolic behavior of PA strains with statistically significant effect of in three strains (ATCC, PA1, and PA4). Smaller NPs size have greater surface reactivity and superior cell penetration capabilities than larger NPs, enabling the release of Zn^2+^^40,66^. The Zn^2+^ released from our 50 nm ZnO NPs could disrupt several essential functional mechanisms of bacteria, including metabolism, enzyme activity and active transport that lead to reduce metabolic activity among the PA strains. By inhibiting these processes, the bacterial cell is unable to function normally. The toxicity of Zn^2+^ stimulates the death of the bacterial cell^37,67^.

We are unaware of large bodies of work evaluating the potential role of ZnO NPs in PA infection, and so our work has some limitations including studies on relatively few PA strains. We conclude that ZnO NPs are a possible disinfectant and antimicrobial agent. This knowledge could be utilized in hospitals to treat nosocomial infection, particularly PA infection. We found that ZnO NPs had significant antigrowth and antibiofilm impacts on PA isolates. ZnO-NPs decreased the expression of genes that cause biofilm and virulence factor production in PA isolates. Our data may be regarded as preliminary and requiring further study. For example the effects of ZnO NPs combined with or containing antibiotics would be of interest. Thus, this study presents preliminary evidence to support the utilization of ZnO NPs as an anti-biofilm QS inhibitor and anti-growth compound relevant to the global challenge of antimicrobial Resistance and antibiotic stewardship, and indicates that further research is required.

## Supplementary Information

Supplementary Information.

## Data Availability

The datasets generated and/or analysed during the current study are available in the Gene Expression Omnibus (GEO) repository, https://www.ncbi.nlm.nih.gov/geo/query/acc.cgi?acc=GSE225492 and Accession Number GSE225492].
