# Supplementary material for: The impact of biosynthesized ZnO nanoparticles from Olea europaea (Common Olive) on Pseudomonas aeruginosa growth and biofilm formation
Source: Sci Rep. 2023 Mar 29;13:5096. doi: 10.1038/s41598-023-32366-1 (PMC10060419; doi:10.1038/s41598-023-32366-1)
Supplement: Supplementary file 1 — Supplementary Information. [file 41598_2023_32366_MOESM1_ESM.docx]

**The Impact of Biosynthesized ZnO Nanoparticles from** **Olea europaea (Common Olive) on Pseudomonas Aeruginosa Growth and Biofilm Formation**

Hafez Al-Momani^a^, Dua’A Al Balawi^b^, Saja Hamed^c^, Borhan Aldeen Albiss ^d^, Muna Almasri^b^, Hadeel AlGhawrie^e^, Logen Ibraheem^b^, Hadeel Al Balawi^b^, Sameer Al Haj Mahmoud^f^, Jeffrey Pearson^g^, Christopher Ward^g^

Table S1: Primers used in assess the expression of quorum sensing genes ^1-3^

**Gene Primer sequence**

lasI F 5′-CGCACATCTGGGAACTCA-3′

R 5′-CGGCACGGATCATCATCT-3′

rhlR F 5′-GCCAGCGTCTTGTTCGG-3′

R 5′-CGGTCTGCCTGAGCCATC-3′

ropD F 5′-CGAACTGCTTGCCGACTT-3′

R 5′-GCGAGAGCCTCAAGGATAC-3

pqsA F 5′-GACCGGCTGTATTCGATTC-3′

R 5′-GCTGAACCAGGGAAAGAAC-3′

pqsR F 5′-CTGATCTGCCGGTAATTGG-3′

R 5′-ATCGACGAGGAACTGAAGA-3′

lasR F 5′-CTGTGGATGCTCAAGGACTAC-3′

R 5′-AACTGGTCTTGCCGATGG-3′

rhlI F 5′-GTAGCGGGTTTGCGGATG-3′

R 5′-CGGCATCAGGTCTTCATCG-3′

lasB F 5′-GGAATGAACGAAGCGTTCTCCGAC-3′

R 5′-TGGCGTCGACGAACACCTCG-3′

Table S2: Interpretavie categories and zone Diameter breakpoint, nearest whole millimeter (mm) for PA ATCC 27853 and the six clinical strains, Abbreviations: ATCC , American type culture collection, I , intermediate, R resistance, S susceptible

|  | Piperacillin (PRL) | Cefepime (FEP) | Ceftazidime (CAZ) | Aztreonam (ATM) | Imipenem (IPM) | Meropenem (MEM) | Gentamicin (CN) | Amikacin (AK) | Levofloxacin (LEV) | Ciprofloxacin (CIP) |
| --- | --- | --- | --- | --- | --- | --- | --- | --- | --- | --- |
| ATCC | 29 (S) | 29(S) | 29(S) | 30(S) | 21(S) | 29(S) | 20(S) | 24(S) | 26(S) | 34(S) |
| PA1 | 42(S) | 36(S) | 34(S) | 40(S) | 35(S) | 44(S) | 20(S) | 19(S) | 44(S) | 48(S) |
| PA2 | 32(S) | 28(S) | 29(S) | 30(S) | 25(S) | 36(S) | 14(I) | 19(S) | 32(S) | 36(S) |
| PA3 | 33(S) | 14(R) | 28(S) | 34(S) | 36(S) | 34(S) | 17(S) | 19(S) | 23(S) | 28(S) |
| PA4 | 25(S) | 25(S) | 25(S) | 24(S) | 18(I) | 35(S) | 29(S) | 29(S) | 21(I) | 40(S) |
| PA5 | 29(S) | 14(R) | 26(S) | 25(S) | 32(S) | 18(I) | 14(I) | 16(I) | 20(I) | 24(I) |
| PA6 | 28(S) | 16R | 24(S) | 23(S) | 17(I) | 19(S) | 13(I) | 18(S) | 16(I) | 22(I) |

Table S3: Biofilm characteristics of the strain used in this study

|  | **Odc** | **OD of positive control** | **2*Odc** | **4*Odc** |  |
| --- | --- | --- | --- | --- | --- |
| ATCC | 0.09 | 1.01 | 0.18 | 0.36 | strong-biofilm producer |
| PA1 | 0.06 | 0.475 | 0.12 | 0.24 | strong-biofilm producer |
| PA2 | 0.06 | 0.764 | 0.12 | 0.24 | strong-biofilm producer |
| PA3 | 0.07 | 0.627 | 0.14 | 0.28 | strong-biofilm producer |
| PA4 | 0.08 | 0.676 | 0.16 | 0.32 | strong-biofilm producer |
| PA5 | 0.07 | 0.642 | 0.14 | 0.28 | strong-biofilm producer |
| PA6 | 0.06 | 0.712 | 0.12 | 0.24 | strong-biofilm producer |


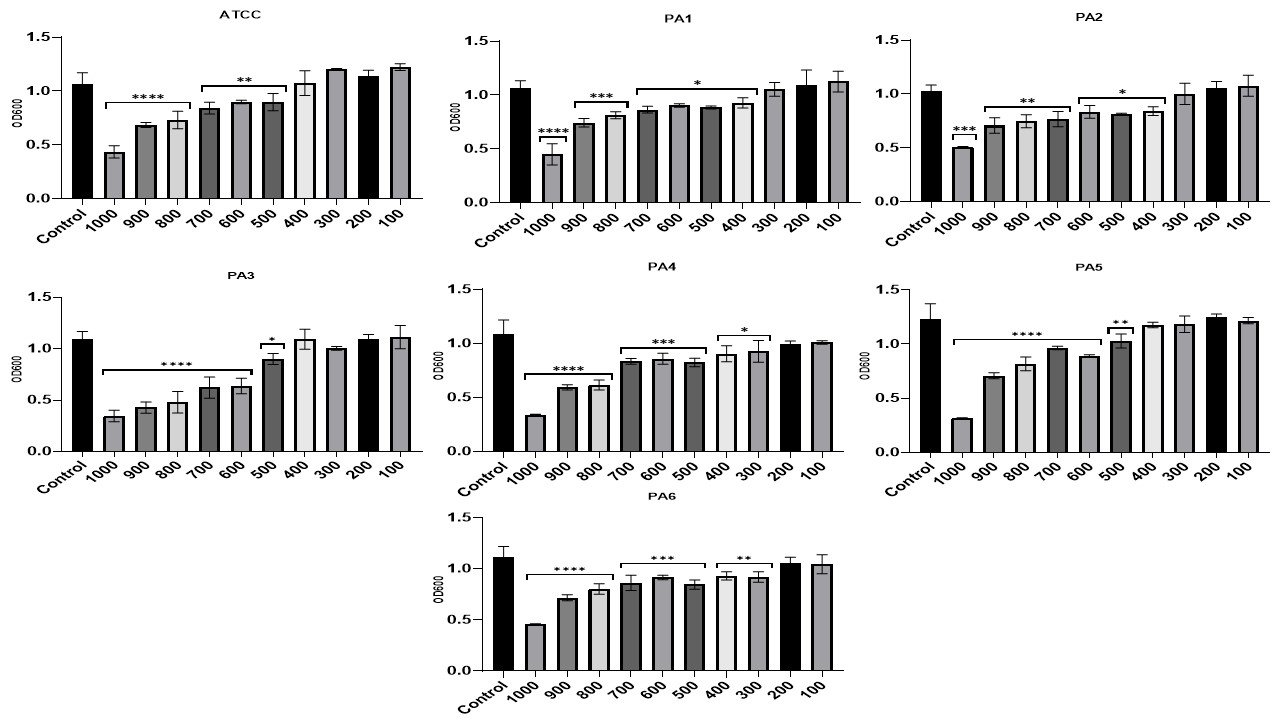


Supplementary Figure 1: The effect of various concentrations (100-1000 µg/ml) of ZnO NPs on the planktonic growth of 1-day mature biofilm after incubation for 24 hours with ZnO NPs


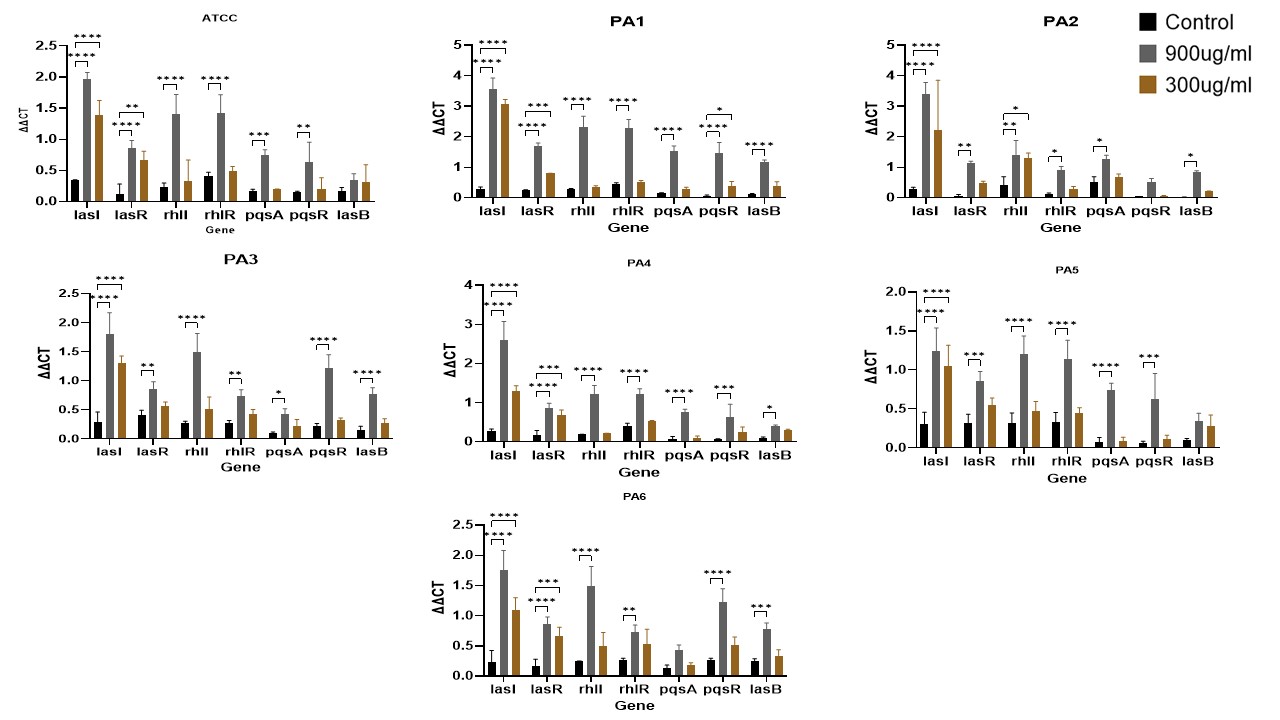


Supplementary Figure 2: The effect of ZnO NPs at concentrations of 900 µg/ml and 300 µg/ml on delta CT values compared to the delta CT values of control samples

1. Naga, N.G., El-Badan, D.E., Rateb, H.S., Ghanem, K.M. & Shaaban, M.I. Quorum Sensing Inhibiting Activity of Cefoperazone and Its Metallic Derivatives on Pseudomonas aeruginosa. *Frontiers in cellular and infection microbiology* **11**, 716789 (2021).

2. Béatrice, J. et al. Relative expression of Pseudomonas aeruginosa virulence genes analyzed by a real time RT-PCR method during lung infection in rats. *FEMS microbiology letters* **243**, 271-278 (2005).

3. El‐Mowafy, S., Shaaban, M. & Abd El Galil, K. Sodium ascorbate as a quorum sensing inhibitor of P seudomonas aeruginosa. *Journal of applied microbiology* **117**, 1388-1399 (2014).
